# Supplementary material for: Rapid and biased evolution of canalization during adaptive divergence revealed by dominance in gene expression variability during Arctic charr early development
Source: Commun Biol. 2023 Aug 31;6:897. doi: 10.1038/s42003-023-05264-5 (PMC10471602; doi:10.1038/s42003-023-05264-5)
Supplement: Supplementary file 2 — Supplementary Information [file 42003_2023_5264_MOESM2_ESM.pdf]

Evolution of canalization: lessons from a classic case of resource polymorphism – Supplementary Figures and Tables

Supplementary Table 1. The first 10 GO terms of genes with maternal patern of expression and of gene with expression biased towards the PL morph in the hybrids, at 200τs.

	GO ID	Term	Annotated genes	Significant genes	Expected	P
**Maternal**	GO:0015917	aminophospholipid transport	14	2	0.03	3.0e-04
	GO:0035999	tetrahydrofolate interconversion	19	2	0.04	5.7e-04
	GO:0070268	cornification	98	3	0.18	8.2e-04
	GO:0070863	positive regulation of protein exit from...	23	2	0.04	8.4e-04
	GO:0097264	self proteolysis	55	2	0.1	4.7e-03
	GO:0045214	sarcomere organization	277	3	0.51	1.5e-02
	GO:0051892	negative regulation of cardioblast diffe...	19	2	0.04	1.6e-02
	GO:0046716	muscle cell cellular homeostasis	105	2	0.2	1.6e-02
	GO:0007517	muscle organ development	1944	7	3.61	1.8e-02
	GO:0003365	establishment of cell polarity involved ...	10	1	0.02	1.8e-02
**PL-biased**	GO:0009799	specification of symmetry	712	2	0.8	4.5e-03
	GO:0010506	regulation of autophagy	951	5	1.07	9.0e-03
	GO:1905209	positive regulation of cardiocyte differ...	141	2	0.16	1.1e-02
	GO:1902477	regulation of defense response to bacter...	10	1	0.01	1.1e-02
	GO:0036306	embryonic heart tube elongation	10	1	0.01	1.1e-02
	GO:0009272	fungal-type cell wall biogenesis	10	1	0.01	1.1e-02
	GO:0060827	regulation of canonical Wnt signaling pa...	11	1	0.01	1.2e-02
	GO:0060577	pulmonary vein morphogenesis	11	1	0.01	1.2e-02
	GO:1990569	UDP-N-acetylglucosamine transmembrane tr...	11	1	0.01	1.2e-02
	GO:0060468	prevention of polyspermy	11	1	0.01	1.2e-02

Supplementary Table 2. Name and location of the genes showing a biased expression in hybrids towards the PL morph, at 150τs and 200τs.

	Symbol	Name	Chromosome
**150τs**			
	LOC111950271	immunoglobulin superfamily DCC subclass member 3-like	LG23
	tp53	tumor protein p53	LG23
	LOC111951066	tumor necrosis factor receptor superfamily member 19	LG23
	LOC111952141	40S ribosomal protein S27-like	LG26
	LOC111956165	immunoglobulin superfamily DCC subclass member 3-like	LG31
	LOC111976118	40S ribosomal protein S27-like	LG16
	adprh	ADP-ribosylarginine hydrolase	LG2
	LOC111980339	tumor necrosis factor receptor superfamily member 19-like	LG20
	LOC111981653	uncharacterized LOC111981653	LG20
	LOC112069817	cyclin-G1-like	Un
	LOC112072678	uncharacterized LOC112072678	Un
	sybl1	synaptobrevin-like 1	Un
**200τs**			
	kera	keratocan	LG24
	LOC111955992	splicing factor U2AF 65 kDa subunit	LG31
	LOC111956824	UDP-N-acetylglucosamine transporter	LG32
	bmp16	bone morphogenetic protein 16	LG4p
	LOC111960782	low choriolytic enzyme-like	LG4p
	LOC111962715	nuclear pore complex protein Nup160-like	LG4q.1:29
	LOC111963418	protein Daple-like	LG4q.2
	LOC111965102	semaphorin-4D-like	LG6.1
	xkrx	XK related X-linked	LG6.2
	zgc:110843	CDGSH iron-sulfur domain-containing protein	LG6.2
	LOC111967380	myozenin-2-like	LG8
	LOC111967383	HLA class II histocompatibility antigen gamma chain	LG8
	LOC111967891	MKL/myocardin-like protein 1	LG8
	LOC111968855	filamin-A-interacting protein 1	LG9
	LOC111969506	switch-associated protein 70	LG10
	LOC111969927	store-operated calcium entry regulator STIMATE	LG11
	fkbp5	FKBP prolyl isomerase 5	LG11
	LOC111972571	vesicular, overexpressed in cancer, prosurvival protein 1	LG14
	mtch2	mitochondrial carrier homolog 2	LG15
	adprh	ADP-ribosylarginine hydrolase	LG2
	LOC111978879	ras-related protein Rab-8A-like	LG19
	LOC111979494	Niemann-Pick C1 protein	LG19
	LOC111981097	parvalbumin-7	LG20
	LOC111982785	claudin-4-like	LG22
	vgll2b	vestigial-like family member 2b	Un
	LOC112069671	trans-Golgi network integral membrane protein 1-like	Un

LOC112069734	calponin-3-like	Un
mybpc2a	myosin binding protein Ca	Un
LOC112072130	transcription factor EB-like	Un
znf106a	zinc finger protein 106a	Un
LOC112075995	ncRNA	Un
LOC112076473	oocyte zinc finger protein XlCOF6-like	Un
LOC112077739	ncRNA	Un
LOC112078614	uncharacterized LOC112078614	Un
SDF2L1	stromal cell derived factor 2 like 1	22
TBX15	T-box transcription factor 15	1
SLC10A7	solute carrier family 10 member 7	4
MLIP	muscular LMNA interacting protein	6

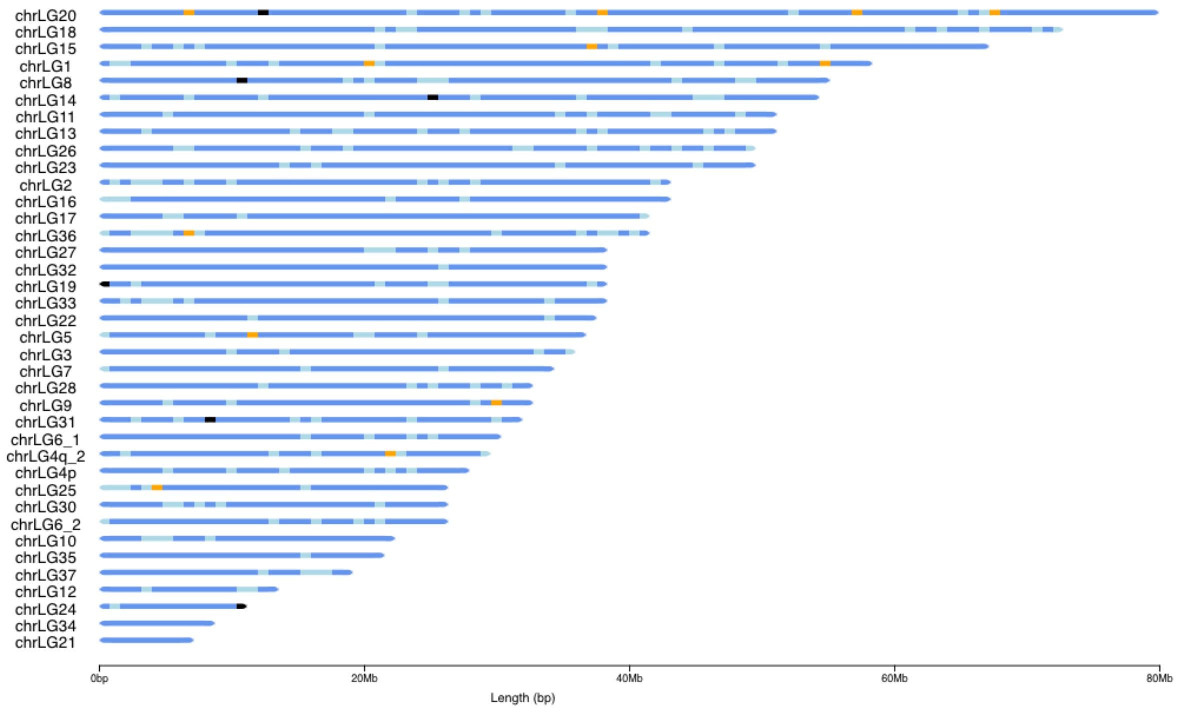

Supplementary Figure 1. Physical location of the genes with differential expression among the two pure morph crosses at $200\tau_s$. Blue: Differentially expressed with no putative dominance pattern. Light blue: No differential expression. Orange: Maternal inheritance, Black: PL-biased expression in hybrids. Uncharacterized chromosomes not shown.

Supplementary Table 3. Rationale to indentify the putative dominance of candidate genes according to the log change and the adjusted P-value.

**Dominance**	**Contrast**	**Log_2 fold change***	**Adjusted P**
**Maternal**	SBxSB vs. PLxPL	<0	<0.1
	SBxPL vs. PLxPL	<0	<0.1
	PLxSB vs. PLxPL	-	>0.1
	SBxPL vs. SBxSB	-	>0.1
	PLxSB vs. SBxSB	>0	<0.1
**PL-dominant**	SBxSB vs. PLxPL	<0	<0.1
	SBxPL vs. PLxPL	-	>0.1
	PLxSB vs. PLxPL	-	>0.1
	SBxPL vs. SBxSB	>0	<0.1
	PLxSB vs. SBxSB	>0	<0.1
**SB-dominant**	SBxSB vs. PLxPL	<0	<0.1
	SBxPL vs. PLxPL	>0	<0.1
	PLxSB vs. PLxPL	>0	<0.1
	SBxPL vs. SBxSB	-	>0.1
	PLxSB vs. SBxSB	-	>0.1
**Additive**	SBxSB vs. PLxPL	<0	<0.1
	SBxPL vs. PLxPL	<0	<0.1
	PLxSB vs. PLxPL	<0	<0.1
	SBxPL vs. SBxSB	>0	<0.1
	PLxSB vs. SBxSB	>0	<0.1
**Overdominant**	SBxSB vs. PLxPL	-	-
	SBxPL vs. PLxPL	>0	<0.1

	PLxSB vs. PLxPL	>0	<0.1
	SBxPL vs. SBxSB	>0	<0.1
	PLxSB vs. SBxSB	>0	<0.1
**Underdominant**	SBxSB vs. PLxPL	-	-
	SBxPL vs. PLxPL	<0	<0.1
	PLxSB vs. PLxPL	<0	<0.1
	SBxPL vs. SBxSB	<0	<0.1
	PLxSB vs. SBxSB	<0	<0.1

* log_2 fold change > 0 corresponds to overexpression in the cross type on left. For simplicity, only log_2 fold change < 0 in SBxSB vs. PLxPL are shown.

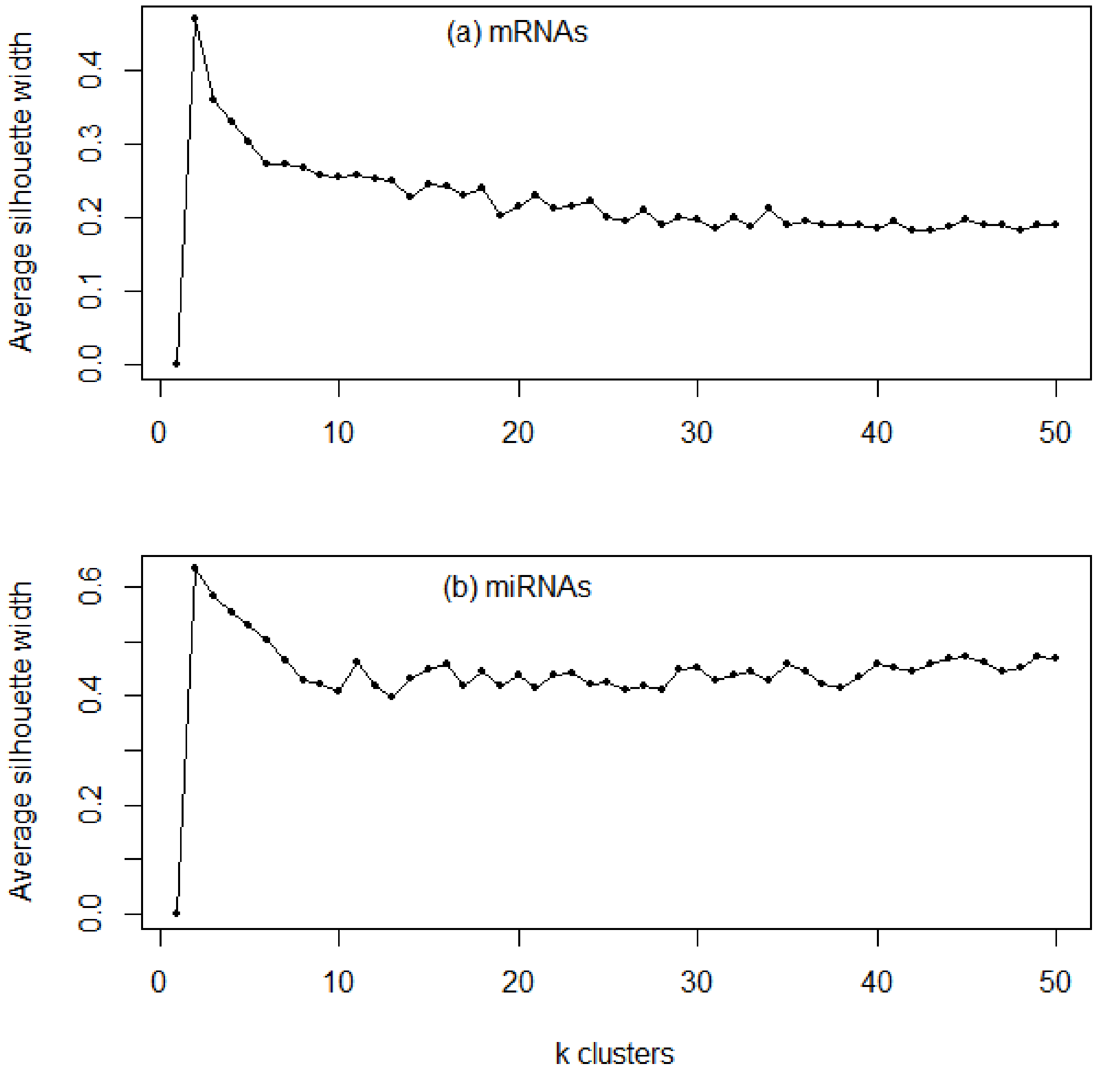

Supplementary Figure 2. Optimal number of clusters for the miRNA dataset. Average silhouette widths for (a) the mRNA dataset and (b) the miRNA dataset.
