# Supplementary material for: Rapid and biased evolution of canalization during adaptive divergence revealed by dominance in gene expression variability during Arctic charr early development
Source: Commun Biol. 2023 Aug 31;6:897. doi: 10.1038/s42003-023-05264-5 (PMC10471602; doi:10.1038/s42003-023-05264-5)
Supplement: Supplementary file 3 — Description of Additional Supplementary Files [file 42003_2023_5264_MOESM3_ESM.pdf]

Description of Additional Supplementary Files

File name: Supplementary Data 1
Description: The first 10 GO terms associated with the mRNAs from each cluster of expression variability.

File name: Supplementary Data 2
Description: The first 10 GO terms of the putative targets of miRNAs from each cluster of expression variability.

File name: Supplementary Data 3
Description: The first 10 GO terms of differential expressed genes at 200τs and for each contrast.

File name: Supplementary Data 4
Description: The first 10 GO terms of the first 5 putative targets of each differentially expressed miRNAs.

File name: Supplementary Data 5
Description: miRNAs in the clusters of coexpression variability and the locations of putative homologs from in situ hybridization data in Wienholds and colleagues, 2005.

File name: Supplementary Data 6
Description: Source data used to plot Figures 2, 3 and 4
