# Supplementary material for: Rapid and biased evolution of canalization during adaptive divergence revealed by dominance in gene expression variability during Arctic charr early development
Source: Commun Biol. 2023 Aug 31;6:897. doi: 10.1038/s42003-023-05264-5 (PMC10471602; doi:10.1038/s42003-023-05264-5)
Supplement: Supplementary file 10 — Reporting Summary [file 42003_2023_5264_MOESM10_ESM.pdf]

Corresponding author(s): Quentin Horta-Lacueva, Zophonías Jónsson , Dagny Thorholludottir, Benedikt Hallgrímsson, Kalina Kapralova

Last updated by author(s): Jul 3, 2023

Reporting Summary

Nature Portfolio wishes to improve the reproducibility of the work that we publish. This form provides structure for consistency and transparency in reporting. For further information on Nature Portfolio policies, see our Editorial Policies and the Editorial Policy Checklist.

Statistics

For all statistical analyses, confirm that the following items are present in the figure legend, table legend, main text, or Methods section.

n/a	Confirmed	
☐	☒	The exact sample size (*n*) for each experimental group/condition, given as a discrete number and unit of measurement
☐	☒	A statement on whether measurements were taken from distinct samples or whether the same sample was measured repeatedly
☐	☒	The statistical test(s) used AND whether they are one- or two-sided<br>*Only common tests should be described solely by name; describe more complex techniques in the Methods section.*
☐	☒	A description of all covariates tested
☐	☒	A description of any assumptions or corrections, such as tests of normality and adjustment for multiple comparisons
☐	☒	A full description of the statistical parameters including central tendency (e.g. means) or other basic estimates (e.g. regression coefficient) AND variation (e.g. standard deviation) or associated estimates of uncertainty (e.g. confidence intervals)
☐	☒	For null hypothesis testing, the test statistic (e.g. *F*, *t*, *r*) with confidence intervals, effect sizes, degrees of freedom and *P* value noted<br>*Give P values as exact values whenever suitable.*
☐	☒	For Bayesian analysis, information on the choice of priors and Markov chain Monte Carlo settings
☒	☐	For hierarchical and complex designs, identification of the appropriate level for tests and full reporting of outcomes
☐	☒	Estimates of effect sizes (e.g. Cohen's *d*, Pearson's *r*), indicating how they were calculated

Our web collection on statistics for biologists contains articles on many of the points above.

Software and code

Policy information about availability of computer code

Data collection	N/A
Data analysis	Code and sources are avaliable/explained in the following GitHub repository: https://github.com/quentin-evo/rna-charr

For manuscripts utilizing custom algorithms or software that are central to the research but not yet described in published literature, software must be made available to editors and reviewers. We strongly encourage code deposition in a community repository (e.g. GitHub). See the Nature Portfolio guidelines for submitting code & software for further information.

Data

Policy information about availability of data

All manuscripts must include a data availability statement. This statement should provide the following information, where applicable:

- Accession codes, unique identifiers, or web links for publicly available datasets
- A description of any restrictions on data availability
- For clinical datasets or third party data, please ensure that the statement adheres to our policy

Raw sequencing data and processed reads are available on the Gene Expression Omnibus, accession GSE193797

Human research participants

Policy information about studies involving human research participants and Sex and Gender in Research.

Reporting on sex and gender	N/A
Population characteristics	N/A
Recruitment	N/A
Ethics oversight	N/A

Note that full information on the approval of the study protocol must also be provided in the manuscript.

Field-specific reporting

Please select the one below that is the best fit for your research. If you are not sure, read the appropriate sections before making your selection.

☐ Life sciences ☐ Behavioural & social sciences ☒ Ecological, evolutionary & environmental sciences

For a reference copy of the document with all sections, see nature.com/documents/nr-reporting-summary-flat.pdf

Ecological, evolutionary & environmental sciences study design

All studies must disclose on these points even when the disclosure is negative.

Study description	We estimated mRNA and micro-RNA (miRNA) expression variability in embryos of sympatric charr morphs and their reciprocal hybrids reared in common garden conditions. The embryos were samples at two developmental stages during the formation of cartilage components of the feeding apparatus. Each biological replicate corresponded to an entire embryo which was sequenced for both mRNAs and miRNAs. We generated 12 families, including 6 families of pure morph crosses and 6 families of reciprocal hybrids, with 3 embryos per families sampled at each of the two time points, summing 72 biological replicates.
Research sample	Arctic charr (Salvelinus alpinus) embryos reared in common-garden conditions, from wild specimens of two sympatric morphs from lake Thingvallavatn, Iceland. The embryos were from F1 from intra-morph and hybrid crosses.
Sampling strategy	We seeked for the maximum sample size given realistic costs for Next-Generation Sequencing data. Among the biological replicates, we maximised the number of families to capture a satisfactory amount of genetic variation.
Data collection	KHK & ZO conducted the sampling of wilf specimens, following by immediate crossing and the rearing of embryos.
Timing and spatial scale	The sampling of wild specimens consisted it several fishing session (gill nets) conducted over the Arctic charr spawning season, in October 2015
Data exclusions	Small RNA sequencing failed for one biological sample. This sample was therefore excluded from the analyses on miRNA expression.
Reproducibility	RNA extraction, sequencing and data prepocessing were conducted following standard protocoles and guidelines. The analyses of gene expression variability were based on an already published algorithm (Simonovsky et al., 2019). The hand written codes and available at https://github.com/quentin-evo/rna-charr
Randomization	The net containing each egg clutch was labelled with a unique code. Three embryos per families were sampled for sequencing and distributed into 3 sequencing batches (miRNA) or sequenced altogether (mRNA).
Blinding	The choice of embryos to sample was pseudo-random since eggs within a single clutch were of the same size and indistinguable. The identity of the samples was recovered using the metadata files during the gene expression analyses.

Did the study involve field work? ☒ Yes ☐ No

Field work, collection and transport

Field conditions	Fishing operations were conducted at dusk and under low wind condiditions (<10m/s). Air temperature was not relevant.
Location	Lake Thingvallavatn, Mjóanes spawning ground. 64.18953359908967, -21.100334736581434

Access & import/export	Sampling of wild fish was conducted by the authors with the permission of the Thingvellir National Park Commission and the owner of the Mjóanes farm.
Disturbance	Fieldwork was conducted aboard a small fishing boat with the same engine type and size format as the ones used by local farmers. Operations on the ground were conducted within the already exhisting facilities (fishing shed) of the Mjóanes farm.

Reporting for specific materials, systems and methods

We require information from authors about some types of materials, experimental systems and methods used in many studies. Here, indicate whether each material, system or method listed is relevant to your study. If you are not sure if a list item applies to your research, read the appropriate section before selecting a response.

Materials & experimental systems

n/a	Involved in the study	
☒	☐	Antibodies
☒	☐	Eukaryotic cell lines
☒	☐	Palaeontology and archaeology
☐	☒	Animals and other organisms
☒	☐	Clinical data
☒	☐	Dual use research of concern

Methods

n/a	Involved in the study	
☒	☐	ChIP-seq
☒	☐	Flow cytometry
☒	☐	MRI-based neuroimaging

Animals and other research organisms

Policy information about studies involving animals; ARRIVE guidelines recommended for reporting animal research, and Sex and Gender in Research

Laboratory animals	Embryos of Salvelinus alpinus, killed before hatching.
Wild animals	Adult Salvelinus alpinus used for extracting gamets. The animal were killed and sampled for various tissues and measurements to serve on multiple research projects.
Reporting on sex	The embryos were sampled before observable sex differentiation.
Field-collected samples	The eggs were reared at approximately 5°C in a hatching tray (EWOS, Norway) under constant water flow and in complete darkness at the Holar University experimental facilities in Verið, Sauðárkrókur. Water temperature was recorded twice a day to estimate the relative age of the embryos.
Ethics oversight	Ethics committee approval is not needed for scientific fishing in Iceland (The Icelandic law on animal protection, Law 15/1994, last updated with Law 157/2012). Rearing of embryos was performed according to Icelandic regulations (licence granted to Hólar University College aquaculture and experimental facilities). HUC-ARC has an operational license accvording to Icelandic law on aquaculture (Law 71/2008), that includes clauses of best practices for animal care and experiments

Note that full information on the approval of the study protocol must also be provided in the manuscript.
